# Prevalence of and factors associated with depression in the hill tribe population aged 40 years and older in northern Thailand

**DOI:** 10.1186/s13033-021-00487-7

**Published:** 2021-06-30

**Authors:** Onnalin Singkhorn, Tawatchai Apidechkul, Khanittha Pitchalard, Katemanee Moonpanane, Pawadee Hamtanon, Rachanee Sunsern, Yosapon Leaungsomnapa, Jintana Thepsaw

**Affiliations:** 1 School of Nursing, Mae Fah Luang University, Chiang Rai Province, Thailand; 2 School of Health Science, Mae Fah Luang University, Chiang Rai Province, Thailand; 3 Center of Excellence for the Hill Tribe Health Research, Mae Fah Laung University, Chiang Rai Province, Thailand; 4 Boromarajonani College of Nursing, Nakhon Si Thammarat, Thailand; 5 Phrapokklao Nursing College, Chanthaburi, Thailand

**Keywords:** Depression, Hill Tribe, Prevalence, Factors associated

## Abstract

**Background:**

Depression is globally recognized as a major mental health problem in all age categories, particularly among those living in poor economic conditions and with low levels of education, including the hill tribe people in northern Thailand.

**Methods:**

This cross-sectional study aimed to estimate the prevalence of depression and determine the factors associated with depression in the hill tribe population aged 40 and over in northern Thailand. Hill tribe people who lived in the selected villages and met the inclusion criteria were invited to participate in the study. A validated questionnaire and the Patient Health Questionnaire-9 (PHQ-9) were used for data collection. An interview was conducted in a private and confidential room in the selected villages between January and April 2019. Logistic regression was used to determine the factors associated with depression at a significance level of α = 0.05.

**Results:**

A total of 601 participants were recruited into the study. More than half (64.23%) were women, 46.76% were Akha, 61.90% were aged 40–59 years, and 76.37% were married. Half of the participants were Christian (57.07%) and had no monthly income (51.25%), and 85% were illiterate. The overall prevalence of depression was 39.10%: 75.74% had mild depression, 17.88% had moderate depression, and 6.38% had severe depression. In the multivariate model, three variables were found to be associated with depression: being female, having a history of substance abuse, and experiencing stress six months prior. Compared to men, women were 2.09 times (95% CI 1.30–3.35) more likely to have depression. Those who had a history of substance abuse were more likely to have depression than those who did not have a history of substance abuse (AOR = 1.97; 95% CI 1.25–3.10). Those who had a history of stress in the prior 6 months were more likely to have depression than those who did not (AOR = 6.43; 95% CI 4.20–9.85).

**Conclusion:**

Public health screening programs to identify depression in the hill tribe population are urgently needed, particularly for women, those who have abused psychoactive substances, and those who have experienced stress.

## Introduction

Depression has been recognized as the largest mental health problem internationally [1]. It is largely and widely reported in individuals in all age categories, from children to elderly adults, who are living in both developed and developing countries [2]. Various factors have been determined to influence the development of depression; these factors vary in different groups of people [1]. The most significant consequence of depression is suicide [3]. The World Health Organization (WHO) reported that more than 264 million people were suffering from depression globally, particularly people aged 40 years and over [1]. Southeast Asia has been ranked the second-highest in the world for depression since 2015 [4]. The WHO reported that depression accounted for 738/100,000 years lived with disability (YLDs) worldwide [4] and estimated that 788,000 people died of suicide related to depression, which accounted for 1.50% of all deaths worldwide [1].

In Thailand, depression has been considered a major mental health problem for the whole population. It has also been reported to occur comorbidly with various physical health problems, particularly in women and elderly populations [1]. In 2016, the Ministry of Public Health in Thailand reported that more than 14 million individuals had been screened for depression in clinics throughout the country; among them, 1.7 million had received psychosocial interventions, and 0.7 million were diagnosed with and treated for depression [5].

Depression impacts individuals at different levels [6]. Individuals who have depression suffer in interactions with family members, friends [7], and community members [8]. Reduced work productivity is another problem for those suffering from depression [7]. Most of the suffering individuals do not present clinical signs or symptoms [1, 2]. Only those who are familiar with and have sufficient information about the disease seek medical care [8, 9]. Given that many individuals suffering from depression are asymptomatic or have mild symptoms, they may not need to seek medical care, particularly in the early stages of depression. However, in the later stages, these individuals may need to be cared for and treated properly [6]. The treatment and care for depression also take time, requiring at least one year for complete treatment, approximately 3 months for intensive treatment, and 6–9 months for continual care and monitoring before the cessation of drugs [6]. In Thailand, depression is associated with high medical costs, and a large number of medical staff specialize in depression care [10]. This is particularly the case in rural areas, which are limited in both public health financial allocation and public health personnel to address the problem, especially in border areas, which are the preferred residential areas of minority and stateless populations [11, 12]. In 2019, the Ministry of Public Health in Thailand reported only 25 community-based psychiatric units, with 0.4 beds per 100,000 population, which is very limited given the overall Thai population of 67 million individuals, including hill tribe people [13].

Most of the hill tribes are located in rural areas and are far from the city, especially along the borders of Myanmar and Laos [10, 14]. In 2019, the WHO estimated 3.5–4.0 million hill tribe people living in Thailand [15]. More than 300,000 hill tribe people lived in Chiang Rai Province in 2019 [10]. The hill tribe people in Thailand are also defined as vulnerable populations with respect to several health problems, including depression due to specific lifestyles, culture, diet and other health behaviors such as methamphetamine and opium use [10, 14]. Most of these individuals live in poor economic conditions and have low levels of education [16]. Currently, a large proportion of hill tribe people are also facing poor access to health care services due to the lack of Thai identification cards (ID cards), which are used for free access to all public services, including medical care and the educational system [17]. Regarding the depression screening instrument, only the Thai version is available through the Thai health care system [18]. All the materials used in mental health clinics are developed for trained clinicians. There is no version of the tool specific for use in the hill tribe people in the peripheral health care unit located in the hill tribe village. Furthermore, there is no scientific information available regarding depression in the hill tribe population. Then, the study aimed to estimate the prevalence of and determine the factors associated with depression among hill tribe people aged 40 years and over by using the high reliability tool for depression detection.

## Methods

### Study design

A cross-sectional study was used to estimate the prevalence of and determine the factors associated with depression among middle-aged and elderly hill tribe populations.

### Study setting

The study setting was 9 hill tribe villages located in the Mae Chan District of Chiang Rai Province in northernmost Thailand. Four different tribes were living in the study setting and were recruited into the project. The inclusion criteria were as follows: hill tribe people aged 40 years and over who had lived in the study area for at least 6 months. However, those who were unable to provide essential information regarding the study protocol, particularly when answering questions related to depression detection, were excluded from the study.

### Study sample

The sample size was calculated based on the standard formula of a cross-sectional design: n = [Z^2^_α/2_ *P*(1 − P)]/e^2^, where Z is the value from the standard normal distribution corresponding to the desired confidence level (Z = 1.96 for 95% CI), P is the expected true proportion, and e is the desired precision. According to the study conducted by Chaiut et al. [9], the prevalence of depression was reported to be 32.9%. Based on Z = 1.96, P = 0.33, 1−P = 0.67, and e = 0.05, at least 339 participants were needed. Adding 20.0% to account for error during the study, at least 408 participants were required for the analysis.

### Research instruments

A questionnaire was developed from the literature review and through discussion with experts in the field to obtain all potential variables related to depression in the hill tribe population. Moreover, intensive and purposive interviews were conducted among 7 health workers who were working in the field and setting, namely, 2 doctors, 3 psychiatrist nurses, and 2 public health staff who were working in mental health clinics in their institute (three from a community health-promoting hospital, three from a district hospital (secondary-level hospital), and one from a provincial hospital (tertiary-level hospital). All information gathered was used for questionnaire development.

There were four parts in the final version of the questionnaire. In part I, 6 questions were used to collect the general information of the participants, such as age, sex, tribe, religion, marital status, and education level. In part II, 6 questions were used to collect information on potential determinants of depression, such as occupation, income, family relationships, psychoactive substance use (which was focused on methamphetamine or opium use), medical conditions such as hypertension and diabetes, daily drug use, and experiencing stress during the six months prior to the interview date. Stress was defined as having a difficult time coping with a problem in daily life, which resulted in trouble sleeping and a decreased appetite. In part III, 20 questions were used to detect knowledge of and attitudes regarding depression and care among the participants. In part IV, a standard form for detecting depression, the Thai version of the Patient Health Questionnaire-9 (PHQ-9), which has a reliability of 0.79 [19], was used to assess depression among the participants.

The reliability and validity of the questionnaire were assessed by different methods. The item-objective congruence (IOC) technique was used for content validity by three external experts who were working in the field: psychiatrists, phycologists, and psychiatric nurses. All experts provided comments to improve the quality of the questions. Afterward, a pilot test was conducted among 15 participants who had characteristics that were similar to those of the study population. A Cronbach’s alpha of 0.74 was obtained for sections of knowledge and attitudes.

### Study procedures

The 9 hill tribe villages were selected by a random method from among the 31 hill tribe villages located in Mae Chan District, Chiang Rai Province, Thailand. The district government officer granted the villages permission to participate in the study. Afterward, the village headmen were provided with all essential details regarding the study. Approximately 5 days prior to the researching arriving at the village, the village headmen informed the residents about the study and made appointments for interviews for all villagers who met the inclusion criteria. On the date of the interview, all participants were provided with all essential information, including the objectives of the study, to ensure that they clearly understood the project.

The consent form was obtained before the initiation of the interview in a private and confidential room, which was prepared by the village headmen (in the village hall). For participants who could not speak Thai, trained public health volunteers were asked to help them complete the questionnaire. The interview took approximately 30 min for each participant.

### Data analysis

Data were double-entered into an Excel sheet before being transferred into the SPSS version 24 (SPSS, Chicago, IL) program for analysis. Categorical and continuous data were analyzed properly to present the characteristics of the participants. The chi-square test was used to compare characteristics between people with and without depression. Logistic regression was used to detect the factors associated with depression at a significance level of α = 0.05.

## Results

More than half of the participants were females (64.23%), 46.76% were Akha, 61.90% were aged 40–59 years, and 76.37% were married. Half of the participants were Christian (57.07%), had no monthly income (51.25%), and had debt (59.07%). Most participants were illiterate and had a primary school education (95.67%), lived with family members (94.01%), had no history of substance use (61.40%), did not smoke (68.22%), and did not use alcohol (79.70%).

With respect to depression, one-third of the participants had a depression (39.10%): 75.74% had mild depression, 17.88% had moderate depression, and 6.38% had severe depression. In comparisons between participants who had depression and those who did not, 12 variables were found to be statistically significant: sex (p-value = 0.035), tribe (p-value < 0.001), religion (p-value < 0.001), family debt (p-value = 0.002), history of psychoactive substance use (p-value = 0.014), smoking (p-value = 0.027), medical condition (p-value < 0.001), stress during the past 6 months (p-value < 0.001), reduction in ability to complete housework (p-value < 0.001), living independently with no need for interaction with people (p-value < 0.001), reduced work productivity within the previous 6 months (p-value < 0.001), and knowledge (p-value < 0.001) (Table 1).

**Table 1:** Comparisons of participants with and without depression

Characteristics	n (%)	Depression		χ^2^	p-value
		Yes	No		
Total	601 (100.00)	235 (39.10)	366 (60.90)	N/A	N/A
Sex					
Male	215 (35.77)	72 (33.49)	143 (66.51)	4.42	0.035*
Female	386 (64.23)	163 (42.22)	223 (57.78)		
Tribe					
Akha	281 (46.76)	84 (29.89)	197 (70.11)	19.48	< 0.001*
Lahu	201 (33.44)	92 (45.77)	109 (54.23)		
Mien	66 (10.98)	34 (51.52)	32 (48.48)		
Lisu	53 (8.82)	25 (47.17)	28 (52.83)		
Age (years)					
40–59	372 (61.90)	146 (39.25)	226 (60.75)	0.01	0.926
≥ 60	229 (38.10)	89 (38.86)	140 (61.14)		
(max = 40, min = 88, mean = 56.91, SD = 10.34)					
Religion					
Buddhist	258 (42.93)	124 (48.06)	134 (51.94)	15.24	< 0.001*
Christian	343 (57.07)	111 (32.36)	232 (67.64)		
Marital status					
Single	142 (23.63)	64 (45.07)	78 (54.93)	2.78	0.095
Married	459 (76.37)	171 (37.25)	288 (62.75)		
Education					
Not educated beyond primary school	575 (95.67)	222 (38.61)	353 (61.39)	1.35	0.244
High school and higher	26 (4.33)	13 (50.00)	13 (50.00)		
Having an income					
No	308 (51.25)	125 (40.58)	183 (59.42)	0.58	0.445
Yes	293 (48.75)	110 (37.54)	183 (62.46)		
Family debt					
No	246 (40.93)	78 (31.71)	168 (68.29)	9.56	0.002*
Yes	355 (59.07)	157 (44.23)	198 (55.77)		
Living situation					
With family member	565 (94.01)	219 (38.76)	346 (61.24)	0.45	0.498
Alone	36 (5.99)	16 (44.44)	20 (55.56)		
History of substance use					
No	369 (61.40)	130 (35.23)	239 (64.77)	6.01	0.014*
Yes	232 (38.60)	105 (45.26)	127 (54.74)		
Smoking					
No	410 (68.22)	148 (36.10)	262 (63.90)	4.88	0.027*
Yes	191 (31.78)	87 (45.55)	104 (54.45)		
Alcohol use					
No	479 (79.70)	183 (38.20)	296 (61.80)	0.79	0.372
Yes	122 (20.30)	52 (42.62)	70 (57.38)		
Having a medical condition					
No	257 (42.76)	79 (30.74)	178 (69.26)	13.18	< 0.001*
Yes	344 (57.24)	156 (45.35)	188 (54.65)		
Experiencing stress 6 months prior					
No	418 (69.55)	101 (24.16)	317 (75.84)	128.65	< 0.001*
Yes	183 (30.45)	134 (73.22)	49 (26.78)		
Reduction in ability to complete housework					
No	497 (82.70)	160 (32.19)	337 (67.81)	57.56	< 0.001*
Yes	104 (17.30)	75 (72.12)	29 (27.88)		
Living independently with no need for interaction with people					
No	534 (88.85)	181 (33.90)	353 (66.10)	54.52	< 0.001*
Yes	67 (11.15)	54 (80.60)	13 (19.40)		
Reduced work productivity within previous 6 months					
No	478 (79.50)	144 (30.13)	334 (69.87)	79.024	< 0.001*
Yes	123 (20.50)	91 (73.98)	32 (26.02)		
Knowledge					
Low	405 (67.38)	136 (33.58)	269 (66.42)	17.24	< 0.001*
Moderate	111 (18.47)	60 (54.05)	51 (45.95)		
High	85 (11.14)	39 (45.88)	46 (54.12)		
Attitude					
Poor	394 (65.56)	151 (38.32)	243 (61.68)	0.89	0.638
Neutral	129 (21.46)	55 (42.64)	74 (57.36)		
Good	78 (12.97)	29 (31.18)	49 (62.82)		

^*^Significance level at α = 0.05

In the univariable analysis, ten variables were found to be associated with having depression: sex, tribe, religion, family debt, history of substance use, having a medical condition, smoking, experiencing stress six months prior to the date of interview, living independently with no need for interaction with people, and reduced work productivity within the previous 6 months (Table 2).

**Table 2:** Univariable and multivariable analyses identifying factors associated with depression in the participants

Characteristics	OR	95% CI	p-value	AOR	95% CI	p-value
Sex						
Male	1.00			1.00		
Female	1.45	1.03 – 2.06	0.036*	2.09	1.30 – 3.35	0.002*
Tribe						
Akha	1.00					
Lahu	1.98	1.36—2.89	< 0.001*			
Mien	2.49	1.44 – 4.30	0.001*			
Lisu	2.09	1.15 – 3.80	0.015*			
Age (years)						
40–59	1.00					
≥ 60	0.98	0.70 – 1.38	0.926			
Religion						
Buddhist	1.93	1.39 – 2.70	< 0.001*			
Christian	1.00					
Marital status						
Single	1.00					
Married	1.38	0.94 – 2.02	0.096			
Education						
Not educated beyond primary school	1.77	0.38–3.66	0.078			
High school and higher	1.00					
Having an income						
No	1.14	0.82 – 1.58	0.445			
Yes	1.00					
Family debt						
No	1.00					
Yes	1.71	1.22 – 2.40	0.002*			
Living situation						
With family member	1.00					
Alone	1.77	0.79–3.66	0.676			
History of psychoactive substance use						
No	1.00			1.00		
Yes	1.52	1.09 – 2.13	0.014*	1.97	1.25 – 3.10	0.003*
Smoking						
No	1.00					
Yes	2.21	1.04–2.09	0.027*			
Alcohol use						
No	1.00					
Yes	1.20	0.80–1.79	0.372			
Having a medical condition						
No	1.00					
Yes	1.87	1.33 – 2.63	< 0.001*			
Experiencing stress 6 months prior						
No	1.00			1.00		
Yes	8.58	5.77 – 12.76	< 0.001*	6.43	4.20 – 9.85	< 0.001*
Reduction in ability to complete housework						
No	1.00					
Yes	1.77	0.89–2.66	0.081			
Living independently with no need for interaction with people						
No	1.00					
Yes	8.10	4.43–15.23	< 0.001*			
Reduced work productivity within the previous 6 months						
No	1.00					
Yes	6.59	4.41–10.32	< 0.001*			
Knowledge						
Low	2.11	0.71–4.66	0.399			
Moderate	1.89	0.51–3.95	0.067			
High	1.00					
Attitude						
Poor	0.97	0.77–4.41	0.321			
Neutral	1.13	0.5505.17	0.171			
Good	1.00					

^*^Significance level at α = 0.05

In the multivariable analysis, three (3) variables remained associated with having depression in the hill tribe population aged 40 years and over: female sex, having a history of substance use, and experiencing stress 6 months prior. Compared to males, females were 2.09 times (95% CI 1.30–3.35) more likely to have depression. Those who had a history of substance use were more likely to have depression than those who did not (AOR = 11.97; 95% CI 1.25–3.10), and those who had experienced stress 6 months prior were more likely to have depression than those who did not (AOR = 6.43; 95% CI 4.20–9.85) (Table 2).

## Discussion

The hill tribes in Thailand live in poor economic conditions and have low levels of education. People aged 40 years and over are facing a high prevalence of depression (39.10%), and a greater proportion of women than men have depression. Among the hill tribe people, those who had a history of substance use and those who experienced stress in the 6 months prior to the interview had a greater chance of having depression than those who did not abuse substances and who had experienced no significant stress in the previous six months.

In our study, it was found that the overall prevalence of depression, which was assessed by the PHQ-9, was 39.10%. Females (42.22%) accounted for a greater proportion of those with depression than did males (33.48%). This finding was consistent with the results of a study conducted in India that reported a depression prevalence of 56.30% in females and 33.70% in males [20]. A systematic review conducted in China in 2017 reported that the prevalence of depression was 27.00%. A study in Croatia reported that the prevalence of depression among people attending primary care clinics was 25.5% [21]. A meta-analysis conducted in the United Arab Emirates found a 12.5–28.6% prevalence of depression [22]. A 2018 study in Thailand among the hill tribe elderly population reported that the prevalence of depression was 32.9% [9]. However, the WHO estimated that the prevalence of depression was 4.40% in the general population, and there were various rates in different age categories [4]. Depression has been increasingly reported from 2005 to 2019 among individuals in all age categories, particularly in developing countries, including Thailand [4].

In our study, we also found that women were at a greater risk than men of having depression in the hill tribe population aged 40 years and over. Several previous studies reported similar results, namely that women are at a higher risk than men of having depression [23–25]. A systematic study [23] clearly demonstrated that depression was more common in women than men in many countries worldwide. Kendler et al. [24] explained the pathway of factors influencing major depression by sex and among twins and found that women had much more complicated pathways and factors related to the development of depression than men. Moreover, the WHO reported that women were more vulnerable to depression than men for several reasons, such as family economic constraints, male dominance in social roles in some communities, sex inequity and lifestyle [25].

Substance abuse was found to be associated with depression in the hill tribe population aged 40 years and over in this study. This finding coincides with the results of a study in the United States that reported that people who abused substances were at greater risk of developing depression [26]. Another study in the United States also demonstrated that substance abuse was significantly associated with depression, particularly among adolescents [27]. Studies in Ethiopia have shown that alcohol use [28] and smoking [29] are associated with the development of depression in adult populations.

Moreover, in our study, stress was found to be significantly associated with depression in the hill tribe population. This finding coincides with those of a meta-analysis by Yang et al. [30], which showed that stress was a major factor contributing to depression in multiple countries, particularly in the adult female population. Moreover, a four-year longitudinal study clearly demonstrated that mental problems, including stress, could significantly affect depression in Canada [31].

We have also identified some limitations of this study. First, due to the hill tribe context in Thailand, they have no previous understanding, concepts or messages related to depression in their life and culture. Therefore, assessing depression by a standard tool developed in other populations may not reflect the real situation. However, with the available tools and existing information regarding depression among hill tribe people, reduced work productivity within the 6 months prior to data collection and a reduction in the ability to complete housework were both found to be associated with depression. Initially, these two new variables were obtained from a group discussion among the hill tribe people living in the village prior to data collection on a large scale. Therefore, these variables need to be carefully considered to determine whether they could be factors associated with the onset of depression among hill tribes using a more robust study method in the future.

During data collection, to ensure that we had obtained highly validated information from the participants, a psychiatric nurse who was a member of the research team used several techniques to provide information relevant to depression to all participants before coming up with the specific questions on the PHQ-9 to identify depression in this study. However, the validity of the PHQ-9 was not determined before its use in the field. Second, when asking questions about “stress in the previous 6 months”, it is difficult to obtain a valid answer from hill tribe people. Although the term “stress” is much more familiar to the hill tribes than “depression”, we do not have a standard to detect stress in the previous six months. Thus, the association between stress in the previous six months and depression needs to be detected with a better study design in the future. Third, based on four different hill tribe cultures, the Lahu have greater difficulty obtaining information, particularly in the aspect of mental health problems; they are not familiar with talking to people outside the village or to people who do not speak their native language. Fourth, some people thought that depression developed from intrafamily member conflicts and as a consequence, family members could not speak to people outside the family. Fortunately, all information obtained from the participants was adjusted and improved through data collection by research team members who were psychiatrists and psychologists before the analysis and interpretation. Last, when performing a cross-sectional study, it is very difficult to interpret the findings in the sense of a causal relationship between the exposures (the independent variables) and depression (the outcome) because these variables are measured at the same point in time. Therefore, further investigation to confirm the associations should be considered.

## Conclusion

Hill tribe people aged 40 years and over are encountering a serious problem of depression, especially those who are women, have stress and have a history of psychoactive substance abuse. Due to their living conditions, including their lack of education and low economic status, the hill tribes in Thailand are highly vulnerable to depression. However, to obtain valid information on depression among the hill tribe people, a highly specific and sensitive screening tool for depression, particularly in the hill tribe context, needs to be developed. A health care system is also needed to extend the service to provide proper care and monitoring for those who are found to have depression. Finally, public health interventions that focus on improving knowledge about depression among hill tribe people and developing a proper channel or method for transferring the knowledge related to depression to the hill tribe people using the local hill tribe languages is strongly recommended.

## Data Availability

Not applicable.

## Abbreviations

CI: Confident interval
ID card: Identification card
IOC: Item-objective congruence
PHQ-9: Patient Health Questionnaire-9
WHO: World Health Organization
YLD: Years Lived with Disability
